# Searching Choices: Quantifying Decision‐Making Processes Using Search Engine Data

**DOI:** 10.1111/tops.12207

**Published:** 2016-06-01

**Authors:** Helen Susannah Moat, Christopher Y. Olivola, Nick Chater, Tobias Preis

**Affiliations:** ^1^ Behavioural ScienceWarwick Business SchoolUniversity of WarwickCoventryCV4 7ALUK; ^2^ Tepper School of BusinessCarnegie Mellon University5000 Forbes Ave., Posner HallPittsburghPA 15213USA

**Keywords:** Search engine data, Google, Wikipedia, Decision making, Behavioral science, Computational social science

## Abstract

When making a decision, humans consider two types of information: information they have acquired through their prior experience of the world, and further information they gather to support the decision in question. Here, we present evidence that data from search engines such as *Google* can help us model both sources of information. We show that statistics from search engines on the frequency of content on the Internet can help us estimate the statistical structure of prior experience; and, specifically, we outline how such statistics can inform psychological theories concerning the valuation of human lives, or choices involving delayed outcomes. Turning to information gathering, we show that search query data might help measure human information gathering, and it may predict subsequent decisions. Such data enable us to compare information gathered across nations, where analyses suggest, for example, a greater focus on the future in countries with a higher per capita GDP. We conclude that search engine data constitute a valuable new resource for cognitive scientists, offering a fascinating new tool for understanding the human decision‐making process.

## Abstract 2

When making a decision, humans consider two types of information: information they have acquired through their prior experience of the world, and further information they gather to support the decision in question. We show that statistics from Google search engine queries can help us estimate the statistical structure of prior experience; and, specifically, we outline how such statistics can inform psychological theories concerning the valuation of human lives, or choices involving delayed outcomes. We conclude that search engine data constitute a valuable new resource for cognitive scientists, offering a fascinating new tool for understanding the human decision making process.

## 1 Introduction

Our increasing interactions with the Internet are creating a digital reflection of modern human life. Events across the world are instantly reported online by ordinary citizens on social media services such as *Twitter*, and a wealth of further information has been uploaded to the World Wide Web. Search engines such as *Google* act as a gateway to this vast new information resource. In the process of making this information available to us, *Google* logs both what content exists online and what content people search for.

Cognitive scientists such as Herbert Simon (1955) have long recognized that when faced with a new situation and a need to act, humans can draw on two sources of information. First, they can consult information they have acquired through their experience of the world to date. Second, at a cost of time and effort, they can gather new information relevant to the question in hand.

Here, we argue that search engine data sets offer intriguing insights into how humans make decisions, by providing us with new methods to model and measure both of these information sources. In the first part of this article, we present results suggesting that analyses of content available online can help us model the information that humans may have previously acquired and stored in memory. In the second part, we show that data on queries made to search engines and other online information resources may provide intriguing new measurements of how we gather information before taking actions in the real world, allowing us to map information gathering processes across the globe. We conclude that search engine data constitute a valuable new resource for investigating the human decision‐making process.

## 2 Using the frequency of content on the Internet to approximate the contents of memory

Human decision making is influenced by memory retrieval and information search processes that fundamentally depend on, and reflect, the statistical structure of the social and economic environment (Stewart, 2009; Stewart, Chater, & Brown, 2006). Consequently, understanding and modeling decision making requires that we quantify the structure of the environment, to better inform theories of human choice. Fortunately, so‐called big data on the Internet, including search engines such as *Google*, increasingly provide a good proxy for various aspects of this statistical structure. One source of such big data is the frequency of documents on the Internet, which may reflect the frequency with which people observe certain events or values.

**Figure 1:**
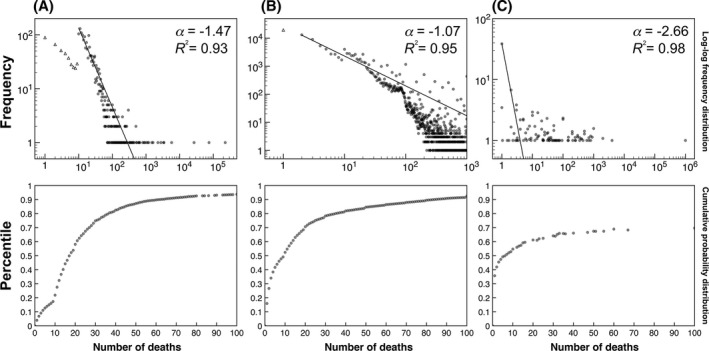
Log–log plots of event‐associated death toll frequency distributions (Top) and their corresponding cumulative probability distributions shown up to 100 deaths (Bottom) (reproduced from Olivola & Sagara, 2009). Solid lines (Top) are best‐fitting power functions of the frequency data, with the associated power parameter estimate (α) and model fit (*R* ^2^) displayed (Top Right). *A*. Fatalities from natural and industrial disasters occurring in 2003 through 2007. *B*. Media attention (in 2000–2007) to mortality‐related events, measured using *Google News Archives*. *C*. Mean recalled event‐associated death tolls occurring in a person's lifetime (as reported by a sample of participants). In all three cases, the distribution of death tolls approximates a power function (as evidenced by the near‐linear shape of their log–log plots). For more details on these figures and the methods used to obtain these data, see Olivola and Sagara (2009).

We illustrate this use of search engine data with an example drawn from Olivola and Sagara (2009), who aimed to approximate the distribution of human losses from deadly events that people likely observe, hold in memory, and can later draw on to evaluate the subjective magnitudes of new deadly events. We refer to these human losses as “event‐associated death tolls.” Olivola and Sagara (2009) first obtained data on the actual distribution of event‐associated death tolls for natural and industrial disasters. Like many distributions in the natural and social sciences (Chater & Brown, 1999; Gisiger, 2001; Kello et al., 2010; Newman, 2005), the frequency of death toll magnitudes resembles a power‐law distribution with a negative exponent, such that small magnitude events (i.e., a few people dying) were much more common than large magnitude events (i.e., large numbers of people dying) (Fig. 1A).

However, the distributions to which people are exposed may not conform to the actual distribution of death tolls. We might expect, for example, an overrepresentation of very large death tolls or the deaths of famous individuals, which could draw disproportionate amounts of media attention.

In an effort to obtain a proxy of the distributions that people might actually observe, Olivola and Sagara (2009) iteratively searched the *Google News Archives* website^1^ to quantify attention to event‐associated death tolls of various magnitudes in the headlines. Data on media attention to event‐associated death tolls were obtained by searching the *Google News Archives* for news articles whose titles contained keywords related to losses of human life (e.g., “10 people died”), while varying the number of deaths being reported (e.g., “10 people died” vs. “11 people died”). For each search, the number of articles (“hits”) reporting a given death toll was recorded, thus measuring of the total media attention allocated to events associated with a given loss in human lives.

Olivola and Sagara (2009) found that the distribution of *Google News Archives* hits also approximated a power‐law distribution (Fig. 1B), although with a shallower slope. This indicates that very large death tolls receive more coverage in the written media, as one would expect. Nonetheless, small death tolls made up a much larger share of the reporting, even when ignoring celebrity deaths.

The question remains whether the subjective distributions that people hold in memory reflect what they see in the media. To examine this question, Olivola and Sagara (2009) asked a group of participants to spontaneously recall events involving human deaths and estimate, for each one, the number of human fatalities. Once again, the contents of memory seemed to follow a power‐law distribution (Fig. 1C), much like the actual distribution and the distribution reflected in the media. In other words, the relative numbers of “hits” returned by *Google News Archives* when searching for deadly events with given death tolls (e.g., “10 people died” vs. “100 people died”) broadly reflect both the objective distributions of death tolls as they actually occur and the subjective distributions stored in people's memory. Olivola and Sagara (2009)'s study illustrates how the contents of the Internet can be used as a proxy for what people observe in their day‐to‐day environment, as well as what they hold in memory—presumably as a result of their observations.

Beyond finding a proxy for the contents of memory, this study showed that people's perceptions of, and reactions to, human fatalities are partly governed by the distribution of death toll magnitudes that they are typically exposed to. Specifically, Olivola and Sagara (2009) applied Decision by Sampling theory (Stewart, 2009; Stewart et al., 2006) to the domain of human life valuation. According to this theory, people evaluate the death toll associated with a specific target event by first drawing upon a sample of comparable events from their memory (e.g., other death tolls they have heard about in the news or from friends) and then comparing the target event‐associated death toll with all those in the sampled set. The psychological “shock” (i.e., the perceived negative value) associated with a target event‐associated death toll is simply the proportion of pair‐wise comparisons in which it is larger than or equal to the comparison death tolls—in other words, its percentile rank among the sampled events.

Olivola and Sagara (2009) outline how many classic findings in the literature on the perception of human losses can be explained as reflecting the distributional properties of human death tolls, following the Decision by Sampling framework. These findings include diminishing sensitivity to human fatalities (Fetherstonhaugh, Slovic, Johnson, & Friedrich, 1997; Friedrich et al., 1999; Slovic, 2007) or risk seeking preferences with regard to human losses (Tversky & Kahneman, 1981). In contrast to purely “descriptive” theories, such as the Weber‐Fechner Law or Prospect Theory (Kahneman & Tversky, 1979), which predict and formalize the concept of diminishing sensitivity but do not specify its underlying processes, Decision by Sampling offers an explanation of its origin: As people are much more exposed to small magnitude death tolls than large magnitude ones, they are more sensitive to a given change in magnitude for small death tolls than for large death tolls. Moreover, unlike the Weber‐Fechner Law or Prospect Theory (which are both “static” theories), Decision by Sampling correctly predicts that manipulating the distribution of death tolls that people consider can influence their perceptions of these death tolls (see Study 2 in Olivola & Sagara, 2009); and, unlike the Weber‐Fechner Law (which, as its name suggests, refers to a universal human tendency), Decision by Sampling correctly predicts that perceptions of death tolls vary across countries according to the distributions of fatalities they experience (see Study 3 in Olivola & Sagara, 2009). Thus, the contents of the Internet can inform theories of human judgment and decision making.

Similarly, data on the frequency distribution of web content allow us to explain and predict people's time preferences (Stewart et al., 2006)—that is, how they trade‐off consumption in the present versus future, and their willingness to forgo a smaller immediate (or sooner) reward for a larger, later one. Stewart et al. (2006) looked at the frequency of occurrence of “hits” generated by *Google* queries for terms related to temporal delays (e.g., “1 day,” “1 year,” etc.; see also Pollmann, 1998; Pollmann & Baayen, 2001). They found that the frequency distribution of duration terms on *Google* also roughly followed a power‐law function. This distribution, when translated through Decision by Sampling theory, predicts that people will exhibit hyperbolic or subhyperbolic delay discounting (i.e., for example, implying that the difference between waiting 1 day vs. 2 days is subjectively much larger than the difference between waiting 101 days vs. 102 days), which corresponds well with the typical findings in the literature (Frederick, Loewenstein, & O'donoghue, 2002; Myerson & Green, 1995; Simpson & Vuchinich, 2000).

Other studies have shown that the contents of the Internet can be used to proxy a broad set of variables that reflect various features of human cognition and human experience. For example, Saiz and Simonsohn (2013) demonstrated a close correspondence between the prevalence of corruption in a geographic location and the relative frequency of documents on the Internet that associate that location with corruption.

In sum, a growing body of research suggests that the frequency distribution of content on the Internet may well reflect the distributional properties of the environment that people are exposed to, and as a result, the contents of human memory. Thus, the Internet offers a useful, powerful, and versatile tool for approximating certain key features of human experience and cognition.

## 3 Using data from search engine queries to measure information‐gathering processes

Data from search engines are not, however, restricted to measuring the statistics of web pages. Search engines such as *Google*, and online resources such as the online encyclopedia *Wikipedia*, also log every request that Internet users across the globe make when searching for information. Both *Google* and *Wikipedia* make this data available to the public at an aggregate level—for example, allowing us to see how often people searched for the word “*restaurant*” or “*recipe*” on *Google* in a given week. If people search for information online to help them make subsequent decisions—for example, where they should eat out, or what they should cook at home—might it be possible to link data on online searches to subsequent real‐world decisions?

To address this question, a dataset describing decisions taken in the real world is required. Vast transactional datasets from financial markets provide exactly such data, recording decisions taken by traders in immense detail. Such large datasets from financial markets are particularly important given their economic impact. Preis, Moat, and Stanley (2013) and Moat et al. (2013) investigated whether links could be found between the frequency with which Internet users searched for financial terms on *Google* and *Wikipedia* and subsequent actions taken in the financial markets. They examined this relationship by implementing a hypothetical trading strategy, in which they bought or sold the Dow Jones Industrial Average (DJIA), depending on changes in how often Internet users had searched for a given term on *Google* (Preis et al., 2013) or viewed a given page on *Wikipedia* (Moat et al., 2013). Where they saw decreases in such online activity in a given week, they bought the DJIA at the beginning of the following week. Conversely, where they saw increases in online activity in a given week, they sold the DJIA at the beginning of the following week.

**Figure 2:**
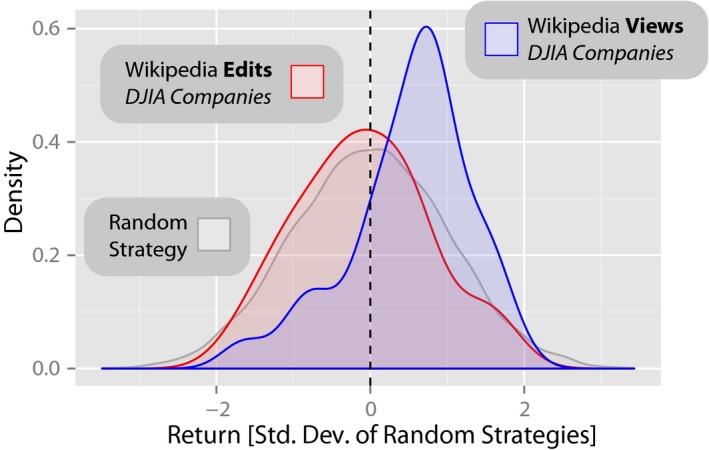
Returns from trading strategies based on *Wikipedia* view and edit logs for articles relating to the companies forming the Dow Jones Industrial Average (DJIA) (reproduced from Moat et al., 2013). The distributions of returns from two portfolios of 30 hypothetical strategies, trading weekly on the DJIA, based on changes in how often the 30 *Wikipedia* articles describing the companies listed in the DJIA were viewed *(blue)* and edited *(red)* during the period from December 2007 to April 2012. The distribution of returns from 10,000 independent realizations of a random strategy is also shown *(gray)*. Whereas random strategies lead to no significant profit or loss, we find that the returns of *Wikipedia* article view‐based strategies for this period are significantly higher than the returns of the random strategies. There is, however, no statistically significant difference between the returns from the *Wikipedia* edit‐based strategies and the random strategies.

Both analyses by Moat et al. (2013) and Preis et al. (2013) suggested that increases in searches for financially related terms tended to be followed by falls in stock market prices. Preis et al. (2013) further demonstrated that words which were classified as more financially relevant, on the basis of the frequency of their occurrence in the Financial Times normalized by their frequency on the Internet as a whole, were more strongly related to subsequent stock market moves. Similarly, Moat et al. (2013) showed that increases in views of *Wikipedia* pages relating to companies listed in the DJIA (Fig. 2), or to more general economic concepts, tended to be followed by stock market drops. No such relationship was found between stock market movements and views of *Wikipedia* pages relating to actors and filmmakers, a topic with no obvious financial connotations.

Together, the results of Moat et al. (2013) and Preis et al. (2013), as well as a further analysis recently reported by Curme, Preis, Stanley, and Moat (2014), suggest that an increase in searches for financially related information online tended to be followed by a fall in stock market prices. What sort of decision‐making mechanism might lead to such a pattern? One potential explanation put forward by Moat et al. (2013) is as follows. Many studies in psychology and behavioral economics have suggested that people are loss averse (e.g., Tversky & Kahneman, 1991; although see Erev, Ert, & Yechiam, 2008); that is, they are more concerned about losing $5 than they are about missing an opportunity to gain $5. Simon (1955) observed that gathering information to support a decision is not cost‐free, incurring at least an expenditure of time and effort. Moat et al. (2013) suggest that humans may be willing to incur higher information gathering costs for decisions that they consider to be of greater consequence. If the decision of greatest consequence that a trader can make is to sell stock at a price lower than the trader believed it was worth, then more information‐seeking efforts may be observed before stock market falls, consistent with Moat et al.'s (2013) and Preis et al.'s (2013) results. Further experiments will be needed to investigate the potential role of loss aversion in information search.

**Figure 3:**
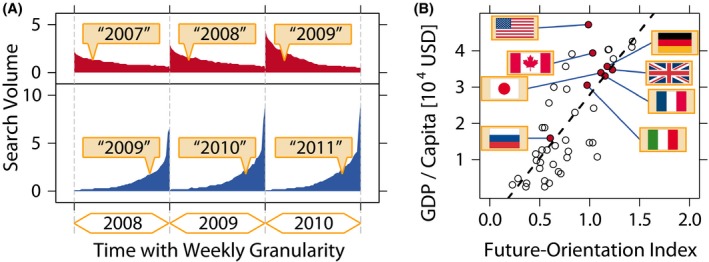
The future orientation index and its correlation with per capita GDP (reproduced from Preis et al., 2012). *A*. The frequency with which *Google* users worldwide search for information about the previous year (in red) and the following year (in blue). Data are plotted for each of 52 weeks per year. The future orientation index for a given year (e.g., 2010) is calculated as the ratio of the total number of searches for the following year (“2011”) to the total number of searches for the previous year (“2009”). *B*. The future orientation index for 45 countries based on searches in 2010, compared with per capita GDP. We demonstrate a strong tendency for countries in which *Google* users enquire more about the future to possess a larger per capita GDP.

A separate study has demonstrated that search engine query data may be used to compare information seeking across the globe, with intriguing differences between countries. Preis, Moat, Stanley, and Bishop (2012) used data on searches for years expressed in Arabic numerals (e.g., “2012”), an almost ubiquitous written representation, to compare search behavior between countries. For the 45 countries with more than 5 million Internet users in 2010, Preis et al. (2012) calculated the ratio of the number of *Google* searches for the coming year (“2011”) to the number of searches for the previous year (“2009”), a quantity which they called the “future orientation index.” Their analyses demonstrated that the future orientation index was strongly correlated with per capita GDP (Fig. 3). More recently, Noguchi, Stewart, Olivola, Moat, and Preis (2014) extended these analyses to show that per capita GDP was also associated with the rate at which national “attention” shifted from the past to the present. Specifically, countries with a higher per capita GDP were characterized by patterns of Internet search that were not only more future focused but also broader in their retrospective temporal horizon (rather than primarily focused on the present or recent past).

One possible interpretation of these results is that differences in attention to the past, present, and future are linked to economic well‐being. A focus on the future and a broader temporal focus may support economic success (Joireman, Sprott, & Spangenberg, 2005; Lynch, Netemeyer, Spiller, & Zammit, 2010; Zimbardo & Boyd, 1999), or conversely, worse socioeconomic conditions may hinder attention to future events and instead keep it focused on immediate or recent events (Holden, Shiferaw, & Wik, 1998; Lawrance, 1991). For example, a focus on the present (a low future orientation) may favor exploiting immediate opportunities, whereas a focus on the future (a high future orientation) may instead favor exploring whether other better options are available (Carstensen, Isaacowitz, & Charles, 1999; for mathematical analysis, see, e.g., Gittins, 1979; Gittins & Jones, 1974). In many environments, there are likely to be substantial long‐term benefits to expending a large amount of time and effort on projects focused on exploration, with potential benefits in the far future. Types of exploration, at the level of individuals and organizations, range from education, to trying out new products, methods, or services, learning new skills, and systematic research and development (e.g., March, 1991). Such exploration tends to lead to long‐term benefits, with positive impacts on the economy. But exploration will be chosen only in the context of long‐term planning; where immediate needs (e.g., concerning the basic provision of food, water, security, and so on) are pressing, the immediate exploitation of existing knowledge is likely to be the dominant behavior (Banerjee & Duflo, 2007). Although such an interpretation can at this stage only be speculative, overall, Preis et al.'s (2012) and Noguchi et al.'s (2014) analyses highlight the opportunities provided by search engine data to measure and compare information gathering at a global scale, a task which was previously near impossible.

A range of studies provide further evidence of links between search engine queries and subsequent decisions (see Moat, Preis, Olivola, Liu, & Chater, 2014, for a summary). For some behaviors, studies demonstrate that search engine data can “predict the present,” measuring real‐world decisions before official data are released (Choi & Varian, 2012). Correlations between search engine query data and real‐world actions span a range of areas, including motor vehicle sales, incoming tourist numbers, unemployment rates, reports of flu and other diseases, and trading volumes in the U.S. stock markets (Askitas & Zimmermann, 2009; Brownstein, Freifeld, & Madoff, 2009; Choi & Varian, 2012; Ettredge, Gerdes, & Karuga, 2005; Ginsberg et al., 2009; Preis & Moat, 2014; Preis, Reith, & Stanley, 2010). In areas where actions may be deliberated for longer, studies show that data on online information gathering can also anticipate future collective behavior. For example, Goel, Hofman, Lahaie, Pennock, and Watts (2010) demonstrated that search query volume predicts the opening weekend box‐office revenue for films, first‐month sales of video games, and chart rankings of songs. Similarly, Méstyán, Yasseri, and Kertész (2013) demonstrate that views and edits of *Wikipedia* pages relating to movies can anticipate their box‐office success on release. This work forms part of a growing literature drawing on data from a range of web services, such as *Twitter* (e.g., Bollen, Mao, & Zeng, 2011), *Flickr* (e.g., Preis, Moat, Bishop, Treleaven, & Stanley, 2013), online news (e.g., Alanyali, Moat, & Preis, 2013; Olivola & Sagara, 2009), and the recommendation website *digg.com* (e.g., Wu & Huberman, 2007) to gain new insights into human behavior (see Bentley, O'Brien, & Brock, 2014, for an overview).

Modern life is now pervaded by massive technological systems, which support our communication, our transport, our retail activities, and much more. Frequent human interaction with these systems is generating increasing amounts of data on large‐scale real‐world behavior (Conte et al., 2012) and thereby steadily increasing the possibilities to link online information gathering with decisions taken in the real world. For privacy reasons, the publicly available data on information requests provided by services such as *Google Trends* and *Wikipedia* only describe behavior at a collective level, rather than an individual level. Access to sequences of search engine requests submitted by individual users—data which are collected by search engines but not made public—would significantly advance our ability to study the information gathering process, such as the heuristics people use to constrain their search for information, or the rules they use to terminate their searches. Barring access to such data, another approach would be to create a new web search interface, perhaps indexing only a limited set of documents, to experimentally collect data and investigate these processes (e.g., Fu & Pirolli, 2007; Pirolli & Fu, 2003). Nevertheless, we argue that these openly available datasets present an unprecedented opportunity for cognitive science to measure a core process in human decision making, both in a natural environment and at immense scale.

## 4 Conclusion

In this article, we have outlined results from a range of previous studies which suggest that data from the Internet can give us new insight into two crucial components of the human decision making process: First, information about the world that a person is likely to have previously observed and stored in memory; and secondly, the process of gathering new information to support a decision. We have shown how data on the frequency with which concepts and events occur on the Internet can help us approximate how often people typically encounter these concepts and events in everyday life. These data can help us understand crucial psychological phenomena, such as how people perceive and react to human fatalities, and how much weight they place on outcomes that occur at different points in the future. We have shown that data on queries made to search engines or online encyclopedias may give us insight into how people gather information before taking decisions. The results outlined suggest that search engine and online encyclopedia data can contribute to our understanding of how information is gathered before stock market falls, and allow us to compare how information is gathered worldwide, where analyses suggest a greater focus on the future and a broader retrospective temporal horizon in countries with a higher per capita GDP. We conclude that data from search engines and other online information resources open up a valuable new avenue for investigating the human decision‐making process, and we call these data to the attention of other cognitive scientists.

## Note

1
*Google News Archive* was an extension of *Google News* that offered access to scanned archives of newspapers and links to other newspaper archives on the web (http://news.google.com/archivesearch). In 2011 *Google News Archives* was discontinued and instead merged with *Google News* (http://news.google.com). In 2014, a modified version of *Google News Archives* was reinstated allowing users to search for articles dating back to 2003.
